# Engineering Sequestration-Based Biomolecular Classifiers with Shared Resources

**DOI:** 10.1021/acssynbio.4c00270

**Published:** 2024-09-20

**Authors:** Hossein Moghimianavval, Ignacio Gispert, Santiago R. Castillo, Olaf B. W. H. Corning, Allen P. Liu, Christian Cuba Samaniego

**Affiliations:** 1 CSHL Course in Synthetic Biology 2022, Cold Spring Harbor Laboratory, Cold Spring Harbor, New York 11724, United States; 2 Department of Mechanical Engineering, University of Michigan, Ann Arbor, Michigan 48109, United States; 3 Chemical Engineering Department, Imperial College London, London SW7 2AZ, U.K.; 4 Department of Biochemistry and Molecular Biology, Mayo Clinic, Rochester, Minnesota 55905, United States; 5 Department of Bioengineering, University of Washington, Seattle, Washington 98125, United States; 6 Department of Biomedical Engineering, University of Michigan, Ann Arbor, Michigan 48109, United States; 7 Department of Biophysics, University of Michigan, Ann Arbor, Michigan 48109, United States; 8 Cellular and Molecular Biology Program, University of Michigan, Ann Arbor, Michigan 48109, United States; 9 Computational Biology Department, Carnegie Mellon University, Pittsburgh, Pennsylvania 15213, United States

**Keywords:** synthetic biology, biomolecular neural networks, molecular sequestration, competitive binding, molecular resource sharing

## Abstract

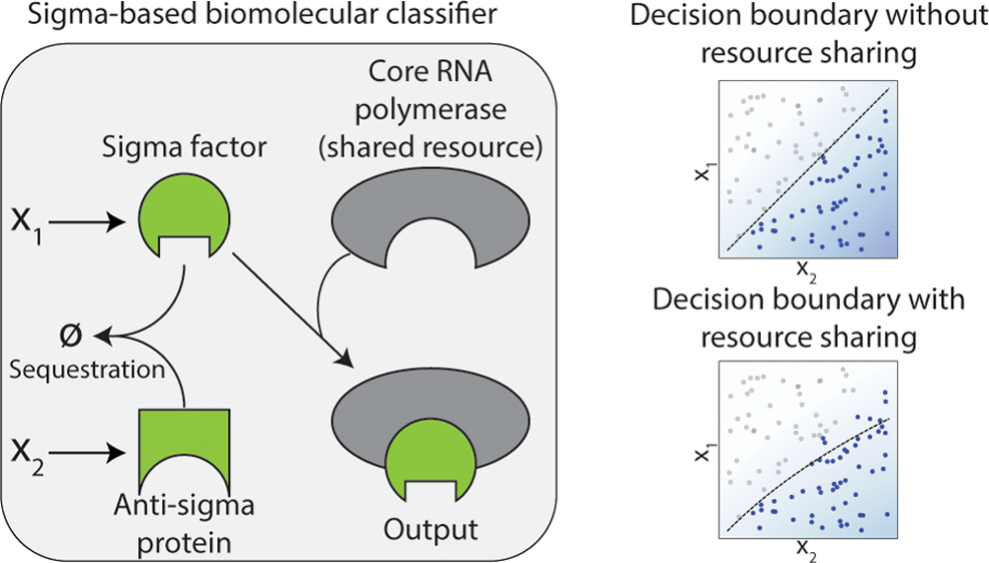

Constructing molecular classifiers that enable cells to recognize linear and nonlinear input patterns would expand the biocomputational capabilities of engineered cells, thereby unlocking their potential in diagnostics and therapeutic applications. While several biomolecular classifier schemes have been designed, the effects of biological constraints such as resource limitation and competitive binding on the function of those classifiers have been left unexplored. Here, we first demonstrate the design of a sigma factor-based perceptron as a molecular classifier working based on the principles of molecular sequestration between the sigma factor and its antisigma molecule. We then investigate how the output of the biomolecular perceptron, i.e., its response pattern or decision boundary, is affected by the competitive binding of sigma factors to a pool of shared and limited resources of core RNA polymerase. Finally, we reveal the influence of sharing limited resources on multilayer perceptron neural networks and outline design principles that enable the construction of nonlinear classifiers using sigma-based biomolecular neural networks in the presence of competitive resource-sharing effects.

## 1 Introduction

Cellular biocomputation is prevalent in nature with examples including activation of genetic circuits during cell proliferation, decision-making in immune response, and a myriad of phosphorylation-based signaling pathways for determining correct response to exogenous signals.^1−3^ The foundation of such computational processes is often laid on molecular interactions, such as protein dimerization or ligand–receptor binding. Thus, the inputs of the computational modules in biological systems are typically the concentration of certain monomeric molecules or ligands, and similarly, the outputs are the concentration of specific dimeric or multimeric molecules.

Drawing inspiration from natural systems, synthetic biologists have been striving to engineer biocomputational schemes in top-down as well as bottom-up synthetic biological systems. While the majority of biocomputational designs rely on utilizing genetic circuits to engineer basic logic gates and simple computational tasks,^4−6^ a few studies have demonstrated engineering protein-based circuits that utilize proteolytic or phosphorylation reactions to generate an output.^7−9^ Although such biocomputational modules enable simple tasks such as biosensing of chemical species and basic computation, they typically generate digital (0, 1 or “on or off”) responses. Furthermore, encoding more sophisticated processing using logic gates demands intricate architectures with many logic gates and computational layers, rendering them convoluted for practical applications in complex tasks.

**Figure 1:**
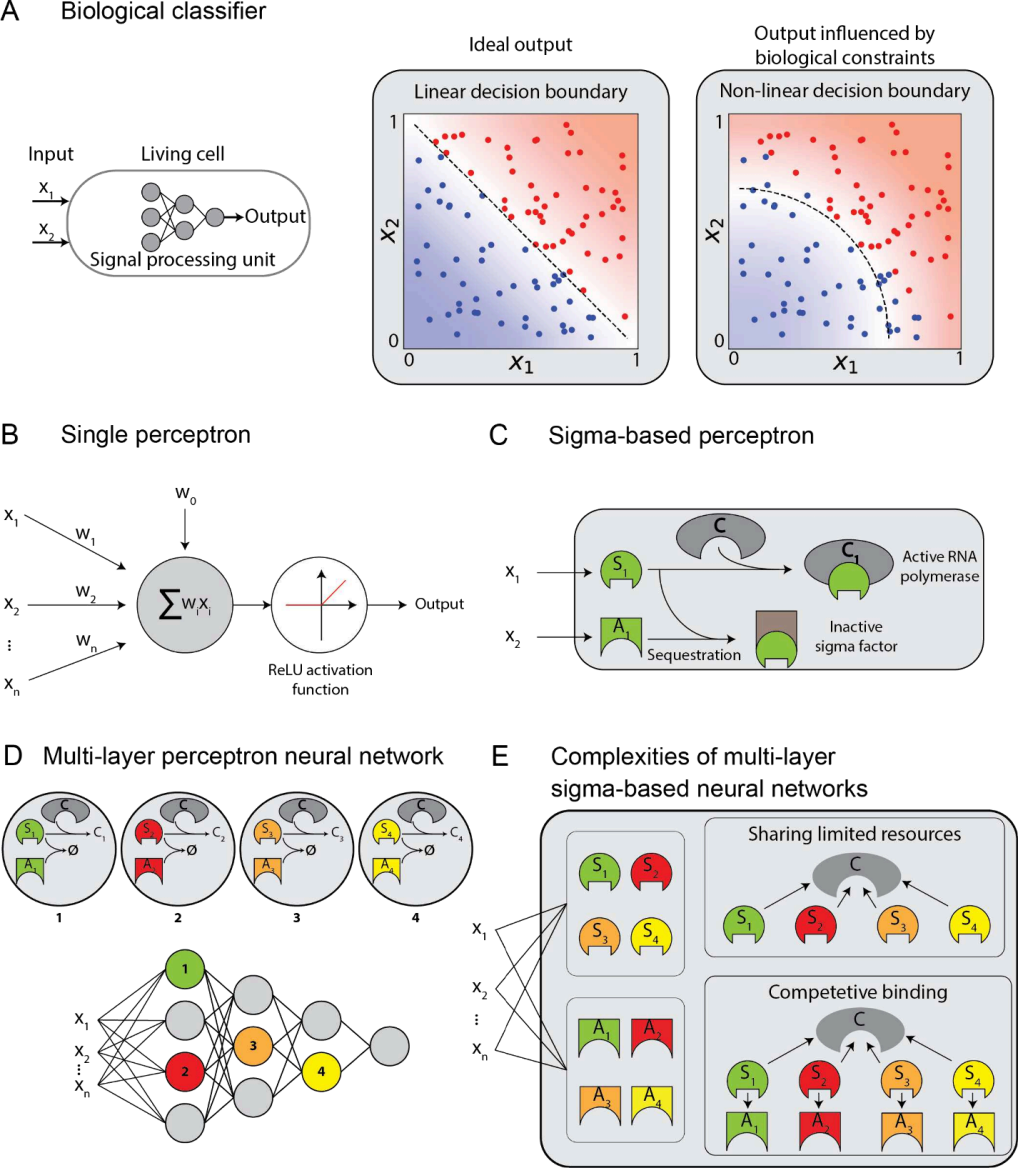
Designing a Biomolecular Neural Network (BNN) utilizing a sigma-based perceptron and multilayer neural networks with shared limited resources for molecular classification: **A**: A molecular classifier as a signal processing unit in living cells enables more sophisticated biocomputation. The resource constraints in biological systems, however, may perturb the designed decision boundary. **B**: The abstract representation of a perceptron as the building block of deep neural networks. Many modern perceptrons utilize the Rectified Linear Unit (ReLU) activation function. **C**: The representation of the biological perceptron design in a sigma-based system using sequestration of a sigma factor and its corresponding antisigma protein. The inputs of the perceptron are concentrations of the sigma and antisigma. **D**: Abstract depiction of multilayer neural networks made of multiple perceptrons in each layer. **E**: Schematic design of a multilayer perceptron in a sigma-based system that poses two major limitations: sharing limited resources and competitive binding. These complexities influence the decision boundary of the multilayer neural network.

Therefore, constructing simple signal processing units inside living systems that can perform intricate computational tasks such as classification and decision-making is of great interest (Figure 1A, left). Implementing molecular classifiers in living cells would enable the creation of ultrasensitive biosensors, programming accurate cellular responses through molecular circuits, and enhanced discrimination of inputs through combinatorial sensing.^10^ For example, a simple linear classifier (Figure 1A, middle) equips a cell with a signal processing system that ideally allows output generation only in certain input regimes (where *x*_1_ and *x*_2_ approach 1 in the example). Further, combining different molecular classifiers results in more complex, nonlinear computation, thus expanding the capabilities of cellular biocomputation.

Binary classification using a linear decision boundary was demonstrated in the field of artificial intelligence (AI) in 1958.^11^ A simple computational unit called “perceptron” performs binary classification by computing the linear combination of weighted inputs and passing the summed weighted inputs through an activation function. The most popular activation function in modern perceptrons is a thresholding function called the Rectified Linear Unit known as ReLU, in which the output is larger than zero when the input crosses a threshold (Figure 1B). The collective processing of inputs by many layers of multiple perceptrons, known as deep neural networks or artificial neural networks (ANNs), can result in the recapitulation of any continuous function,^12^ thus making ANNs capable of performing complicated tasks such as nonlinear classification and accurate prediction.^13−16^

The simple architecture of a perceptron has motivated many efforts toward the creation of a biological perceptron as the signal processing unit for linear classification.^10^ The construction of a single biological perceptron can also pave the way for implementing nonlinear input classification in living cells by utilizing multiple perceptrons, thus creating biomolecular neural networks (BNNs). A biological perceptron must demonstrate the principal characteristics of an ANN (or computer-based) perceptron; i.e., the biological perceptron must include controllable molecular elements that determine the perceptron’s input weights and activation function.

However, as opposed to ANN perceptrons, biological perceptrons face challenges in linear classification due to biological constraints such as limited resources, competitive binding, and nonspecific binding between molecules. Resource constraints in biological systems have been shown to significantly impact the function of biocomputational modules based on molecular interactions.^17−21^ For example, the competition between housekeeping sigma factor RpoD with minor sigma factors such as RpoN, RpoH, and RpoF in *Escherichia coli* (*E. coli*) determines the response of *E. coli* to conditions like nitrogen-deficiency, heat shock, or the need for chemotaxis, respectively, by regulating expression of genes important for growth and survival of bacteria.^22^ Additionally, competitive binding of only a few promiscuous ligands to various receptors has been demonstrated to accommodate a wide range of signaling activities in multicellular organisms.^23^ Similarly, it was recently shown that competitive protein dimerization allows small networks made of monomeric proteins to encode an extensive range of homo- or heterodimeric outputs through precise adjustments in the concentration of network monomers.^24^ Likewise, competition between transcription factors that bind to the same RNA polymerase may affect the output of engineered genetic circuits in bacteria.^18,19,25^ These resource constraints can cause perturbations to the biological perceptron function, thus influencing its decision boundary (Figure 1A, right). Subsequently, BNNs made from the biological perceptron with an altered output will also generate decision boundaries that may not closely follow their ideal design.

A few biological perceptrons have been demonstrated by using inducible gene expression networks,^26−29^ enzymatic processing of different metabolites,^30^ principles of DNA strand-displacement,^31−34^ and DNA-processing enzymes.^35^ Relying on the sequestration of two interacting proteins, a biomolecular classifier with tunable positive and negative weights was designed computationally^36^ and tested experimentally to achieve nonlinear classification in mammalian cells.^29^ Similarly, a phosphorylation-based neural network with positive and negative weights that perform nonlinear classification (i.e., recapitulating XNOR and XOR) was designed by Cuba Samaniego et al.^37^ Recently, a protein-based neural network that achieves linear classification was implemented by exploiting coiled-coil dimerization of engineered peptides.^38^

Although these studies utilize different approaches to create a biological perceptron, they all rely on competitive interactions between input-processing molecules with a shared pool of limited resources (e.g., RNA polymerases, ribosomes,^21,25,39^ coactivators, transcription factors,^40^ and enzymes like Cas proteins^41^ or proteases^42^). However, the effects of resource constraints on the function of these perceptrons have remained unexplored.

Here, we develop a mathematical model to simulate a biological perceptron based on sigma factors—transcription factors which outnumber the RNA polymerase and hence compete for binding to it^18,22,43−45^—that bacteria naturally use to regulate gene expression. Leveraging competitive dimerization between a sigma and either its corresponding antisigma molecule or RNA polymerase, we demonstrate the design of a simple perceptron with a nonlinear activation function capable of realizing positive and negative weights (Figure 1C). We then impose two physiological requirements on our model to account for both competition between the input sigma factor and other present sigma factors as well as the limited available resources (i.e., core RNA polymerase^18,44,45^). We show that resource sharing reveals its effect on the function of the perceptron by suppressing the output, while introducing a slight perturbation to the linear decision boundary. Lastly, since engineering nonlinear decision boundaries require multilayer perceptron networks (as depicted in Figure 1D), we explore designing sequestration-reliant multilayer sigma-based perceptron networks in the presence of perturbations caused by sharing limited resources (Figure 1E). We demonstrate that resource sharing leads to deviations from ideal design that affect the output of the multilayer perceptron network. However, despite the nonideal function of perceptrons due to resource sharing and limited resources, we outline simple design principles for encoding nonlinear response patterns such that they closely resemble their ideal design. Our analyses of biological perceptron and BNN function in the presence of resource constraints can also be utilized to model molecular classifiers *in silico* for more precise prediction or design of outputs of these biocomputational systems.

## 2 Results

Throughout the manuscript, we indicate chemical species with capital letters (e.g., *X*) and their concentration with the corresponding lowercase letters (e.g., *x*). Where possible, reaction and network outputs are normalized throughout the manuscript to allow dimensionless comparison between different conditions. Table 1 at the end of the Results section summarizes all parameters used in the manuscript for mathematical analyses and computational simulations.

**Table 1:** List of Parameters Used in Computational Simulations^a^

Parameter	Symbol	Unit	Value	Figure	ref.
Perceptron input species	[*x*_1_, *x*_2_]	μM	[0 → 1]	2, 3, 4	
Competing perceptron input species	[α, β]	μMh^–1^	[0, 0.5, 1]	2	(59)
Core RNA polymerase (RNApol)	*c*	μM	[0 → 1]	2, 3, 4	
Sigma-antisigma sequestration rate	γ_1_	μM^–1^ h^–1^	*	2	(60,61)
Sigma-RNApol complex formation rate	γ_2_	μM^–1^ h^–1^	10	2	(18,61)
Degradation rate	δ	h^–1^	1	2, 3, 4	(59)
Node 1 input weights	[*w*_1_, *w*_2_]	h^–1^	[1, 1]	3	(59)
Node 2 input weights	[α_2_, β_2_]	h^–1^	[0, 0]	3	
			[0.5, 0.5]	3	
Node 1 input weights	[*w*_0_^1^, *w*_1_^1^, *w*_1_^2^]	h^–1^	[1, 1, 1]	4A	
Node 2 input weights	[−*w*_0_^2^, −*w*_2_^1^, *w*_2_^2^]	h^–1^	[0.5, 1, 1]	4A	
Node 1 input weights	[−*w*_0_^1^, *w*_1_^1^, *w*_1_^2^]	h^–1^	[1.2, 1, 1]	4E	
Node 2 input weights	[*w*_0_^2^, −*w*_2_^1^, −*w*_2_^2^]	h^–1^	[0.7, 1, 1]	4E	
Node 3 input weights	[−*w*_0_^3^, *w*_1_^3^, *w*_2_^3^]	h^–1^	[0.15, 4, 4]	4E	
Node 1 input weights	[*w*_0_^1^, −*w*_1_^1^, −*w*_1_^2^]	h^–1^	[0.4, 0.5, 0.5]	4H	
Node 2 input weights	[−*w*_0_^2^, *w*_2_^1^, −*w*_2_^2^]	h^–1^	[0.8, 1.5, 0.5]	4H	
Node 3 input weights	[−*w*_0_^3^, −*w*_1_^3^, *w*_2_^3^]	h^–1^	[0.8, 0.5, 1.5]	4H	
Node 4 input weights	[*w*_0_^4^, −*w*_1_^4^, −*w*_2_^4^, −*w*_3_^4^]	h^–1^	[0.3, 1, 1, 1]	4H	

a The value of parameters indicated with an asterisk is described in their corresponding figures. The values for rates of production, decay, sequestration, and sigma factor-RNApol binding are determined based on measurements found in the corresponding reference listed in the column ref.

### 2.1 Design of a Rectified Linear Activation Unit (ReLU) Based on Sigma Factor-Antisigma Factor Interaction in the Presence of Shared Limited Resources

**Figure 2:**
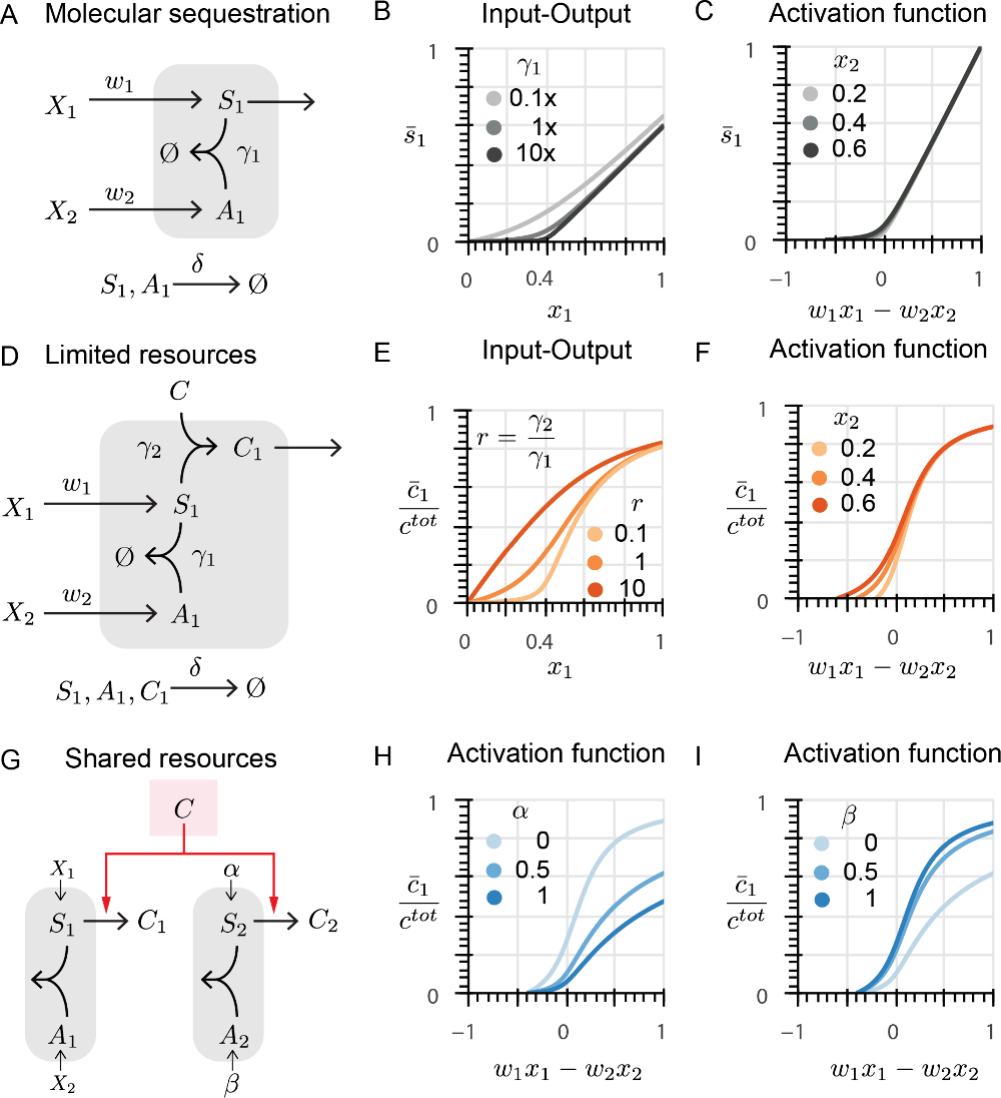
Molecular sequestration of a sigma factor (*S*_1_) and its antisigma (*A*_1_) recapitulates variants of ReLU activation function. **A**: Schematic representation of chemical reactions depicting molecular sequestration of *S*_1_ and *A*_1_. The inputs are concentrations of species *X*_1_ and *X*_2_ that generate *S*_1_ and *A*_1_, respectively. The output is the steady-state concentration of *S*_1_ shown as *s̅*_1_. **B**: The effect of the sequestration rate (γ_1_) on the input–output relationship of the sequestration reaction. **C**: The relationship between *s̅*_1_ and the inputs *x*_1_ and *x*_2_ asymptotically converges to a soft ReLU function in the fast sequestration regime. **D**: Schematic representation of chemical reactions depicting molecular sequestration of *S*_1_ and *A*_1_ as well as the complex formation of *S*_1_ with a limited amount of core RNA polymerase (*C*). The inputs are concentrations of species *X*_1_ and *X*_2_ that generate *S*_1_ and *A*_1_, respectively. The output is steady-state normalized concentration of *S*_1_-*C* dimer (*C*_1_) shown as . **E**: The ratio of the RNApol-*S*_1_ complex formation rate to the sequestration rate (referred to as competitive binding ratio *r*) influences the input–output relationship in the sequestration system. Low *r* values allow the construction of a thresholding function. **F**: In the fast sequestration regime and slow complex formation (*r* → 0 referred to as fast competitive binding regime), the output of the sequestration reactions in the presence of limited resources resembles an asymptotic saturated ReLU (ASReLU). **G**: Schematic representation of chemical reactions depicting molecular sequestration of *S*_1_ and *A*_1_ as well as the complex formation of *S*_1_ with a limited amount of core RNA polymerase (*C*) in the presence of a competing sigma factor (*S*_2_) and its corresponding antisigma (A_2_). The inputs are concentrations of species *X*_1_ and *X*_2_ that generate *S*_1_ and *A*_1_ with rates of *w*_1_ and *w*_2_, respectively. The output is steady-state normalized concentration of *S*_1_-*C* dimer (*C*_1_) shown as . **H**: The effect of concentration of species α that generates the competing sigma factor *S*_2_ on the activation function of the sequestration reaction. The addition of competitive binding reduces the amount of total *C* available, thus lowering the saturation level of the ASReLU function. **I**: The effect of concentration of species (β) that generates *A*_2_ that sequesters the competing *S*_2_ on the activation function of the sequestration reaction. Higher sequestration of *S*_2_ results in a higher concentration of available *C*, thus increasing the saturation level of the ASReLU function.

Molecular sequestration is the stoichiometric binding between two species that results in the formation of a dimeric complex. An example of molecular sequestration is the interaction between sigma factors and their corresponding antisigma proteins that leads to the formation of a complex that is unable to promote gene expression (Figure 1C). Such interaction can be modeled assuming the sigma factor *S*_1_ and antisigma factor *A*_1_ are produced from species *X*_1_ and *X*_2_ at rate constants *w*_1_ and *w*_2_, respectively. Additionally, the produced proteins *S*_1_ and *A*_1_ degrade at rate δ and sequestration occurs with rate constant γ_1_ (as shown in Figure 2A). We summarize the list of chemical reactions as follows:
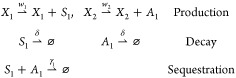


Note that for simplicity, we assume that binding reactions are irreversible. This assumption does not affect the steady-state output of the reactions although it cannot be used to analyze the dynamic behavior of the system (see SI section 3 for derivation of the steady-state output of the system assuming reversible reactions). Depending on the sequestration rate (γ_1_), the output of the system, *s̅*_1_, which stands for the steady-state amount of *S*_1_, follows the input (*w*_1_*x*_1_) in different patterns (Figure 2B). To evaluate two regimes of output at the steady state, we define a dimensionless positive parameter . In the fast sequestration regime (where the binding affinity of *S*_1_ and *A*_1_ is large) when 0 < ξ ≪ 1, modeling the interaction between *S*_1_ and *A*_1_ (see section 1.1 in SI for mathematical derivation) leads to a thresholding function^36^ (a function that generates positive outputs only when the input is larger than a threshold) between the output *s*_1_ and input *x*_1_ shown in eq 1 and depicted in Figure 2B.
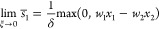
1

Equation 1 describes a nonlinear relationship between *s̅*_1_ and *x*_1_ where *s̅*_1_ is nonzero and proportional to *x*_1_ only when *x*_1_ is larger than a threshold. On the other hand, when ξ ≫ 1, due to the slow kinetics of *S*_1_ – *A*_1_ binding, the relationship between *s̅*_1_ and *x*_1_ becomes nonlinear for the whole range of *x*_1_ and the thresholding behavior is lost (Figure 2B).

The assumption of fast sequestration of a sigma factor by its corresponding antisigma molecule is valid as previous studies on various antisigma molecules have found their rapid effect on transcriptional activity of their target sigma factors.^46−48^ In addition, molecular controllers based on fast sigma-antisigma interaction have been constructed and tested,^49−51^ thereby providing evidence for fast sequestration assumption.

In eq 1, *x*_1_ and *x*_2_ represent the concentration of input species that generate *S*_1_ and *A*_1_ with production rates of *w*_1_ and *w*_2_, respectively. At steady state and a fast sequestration regime, eq 1 converges asymptotically to a ReLU function. Therefore, we named the relationship between *s̅*_1_ with the inputs *x*_1_ and *x*_2_ the Asymptotic ReLU (AReLU) function (Figure 2C). Thus, the sequestration relationship between *S*_1_ and *A*_1_ resembles a simple perceptron with an AReLU activation function and weights of *w*_1_ and −*w*_2_ for inputs *x*_1_ and *x*_2_, respectively. The quasi-linear relationship regime between *s̅*_1_ and *x*_1_ (that resembles a soft ReLU function^52^) indicates that the steady-state available amount of *S*_1_ is simply the difference between the total steady-state amounts of *S*_1_ and *A*_1_, similar to other sequestration-based calculators demonstrated previously.^53^ In other words, the outcome of the sequestration chemical reaction simply calculates the subtraction of total *S*_1_ and *A*_1_ and does not produce any product if *S*_1_ is lower than *A*_1_. However, in slow sequestration, the amount of *s̅*_1_ depends on the input *x*_1_ through a nonlinear relationship without displaying thresholding behavior (Figure 2B).

In natural systems, sigma factors bind to either their corresponding antisigma or the RNA polymerase (RNApol) core. In the latter case, the RNApol-sigma factor complex binds to a specific sigma promoter in the DNA sequence and drives the expression of downstream genes. When free *S*_1_ is present, it binds to the RNApol to initiate transcription. Therefore, when designing sigma-based neural networks, since the amount of RNApol-sigma factor complex directly influences protein expression, the interaction between *S*_1_ and the RNApol core should be considered. Assuming that the sigma factor *S*_1_ binds to a limited amount of available RNApol core *C*, with total concentration denoted as *c*^tot^, at rate γ_2_, the total amount of RNApol-sigma factor complex, denoted as *C*_1_, can be calculated by solving the system of ordinary differential equations (ODEs) representing the following chemical reactions which are schematically illustrated in Figure 2D:
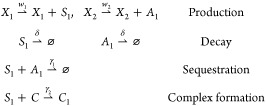


To analyze the input–output relationship of the chemical reactions that consider limited resources, we introduce a dimensionless variable  referred to as competitive binding ratio that represents the ratio of sigma factor-RNApol affinity to the sequestration rate. Solving the ODEs representing the above reactions (see section 1.2 in SI for derivation) reveals that the relationship between steady-state *C*_1_ denoted as *c̅*_1_ (and consequently its normalized value denoted as *c̅*_1_^*n*^ = *c̅*_1_/*c*^tot^) and *x*_1_ depends on the competitive binding ratio. When *r* ≫ 1 (slow competitive binding regime) the input–output relationship deviates from the ideal behavior (thresholding function) observed in Figure 2B. However, when *r* ≪ 1 (fast competitive binding regime), *c̅*_1_ follows the thresholding function (Figure 2E). Hence, when input *x*_1_ is lower than the threshold, there is no response, but when input is higher than the threshold, the response is nonzero and ultimately saturates at 1 (*c*^tot^).

Equation 2 describes the input–output function in the fast competitive binding regime (*r* ≪ 1).
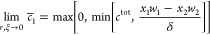
2

Further, eq 2 is closely similar to AReLU, eq 1, with the difference of having a limit on the output (*c*^tot^) which is introduced due to limited resources. Thus, the effect of limited resources, in this case, the total RNApol core, causes the deviation of *c̅*_1_ from a linear trend when *x*_1_ and *x*_2_ vary. Nevertheless, in the fast competitive binding regime even with a limited RNApol core, the activation function still performs as a subtraction calculator, although it is saturated at *c*^tot^ (Figure 2F). Equation 2 characterizes this nonlinear relationship of *c̅*_1_ with inputs *x*_1_ and *x*_2_. We call this activation function asymptotic saturated ReLU (termed ASReLU hereafter). It is worth noting that while the value of *c*^tot^ in *in vitro* systems can be controlled by simply diluting the reaction, *in vivo* regulation of total amount of resources (*C*) could be achieved via inhibition of RNApol activity by a small peptide called Gb2^54^ or overexpression of RNApol subunits β and β′ as described by Izard et al.^55^

Despite ASReLU maintaining its function as a subtractor when the resources are limited, it is rarely the case for substrates to interact with their binding partners without other competing factors. For example, various sigma factors may compete with each other to bind to the available RNApol core molecules.^18,22,43−45^ Thus, when this competition is considered, the function of ASReLU may change. A simplified model consisting of two competing sigma factors (*S*_1_, *S*_2_) and their corresponding antisigma factors can be utilized to predict the function of ASReLU in the presence of competing sigma factors (Figure 2G). For simplicity, here we assume that the competing sigma factors have identical affinities for their corresponding antisigma proteins as well as *C*. The effect of different kinetics is investigated in the next section. The following chemical reactions represent the model:
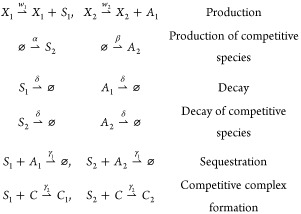


Solving at steady state, the set of ODEs modeling the above equations yields eq 3 (for mathematical derivation, see SI section 1.3), which describes an expression of *c̅*_1_ as a function of inputs (*x*_1_ and *x*_2_) and *c*^tot^. Although it is challenging to find a closed-form expression of *c̅*_1_ at steady state, we can find an expression of *c̅*_1_ as a function of inputs *x*_1_, *x*_2_, and *c̅*_2_ (unknown). This expression is useful to understand the effect of coupling between the two competing sigma factors on their binding to the RNApol. Equation 3 shows that the available RNApol is depleted by the other sigma factor, reflected in the term *c*^tot^ – *c̅*_2_. Additionally, the amount of depletion of available resources will depend on the production rates of both the competing sigma factor and its antisigma protein. Yet, in the fast sequestration regime (ξ → 0), and fast competitive binding (*r* → 0), the system converges to an ASReLU function with a lower saturation magnitude captured by *c*^tot^ – *c̅*_2_.
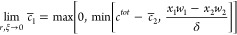
3

Since *c̅*_2_ only influences the ASReLU saturation level, the performance of the ASReLU function in calculating the difference between *S*_1_ and *A*_1_ as a thresholding function is sustained under the effect of a competing sigma factor (Figure 2H). However, the limit of output *c̅*_1_ (the saturation level) further decreases as the amount of competing sigma factor *s*_2_ increases which corresponds with higher α (Figure 2H). This trend reverses back to the noncompeting ASReLU (eq 2) when the amount of antisigma factor *A*_2_ increases with higher β since less *S*_2_ is available to compete with *S*_1_ (Figure 2I).

Overall, molecular sequestration in a sigma-factor-dependent translation system models variations of the ReLU function. In ideal conditions where the RNApol core is unlimited, this dependency is perfectly quasi-linear and reflects the difference between *S*_1_ and *A*_1_, enabling an AReLU function in a fast sequestration regime. However, in the presence of limited as well as shared resources, the trend between the sigma factor-RNApol complex and the differential value of *S*_1_ and *A*_1_ deviates from a linear trend and this deviation becomes more intense as the total amount of free *S*_2_ increases. This relationship in fast sequestration and competitive binding regimes is captured by the ASReLU function. However, since ASReLU still represents the difference of *S*_1_ and *A*_1_, we reasoned that the activation function can be used to create a perceptron, called a sigma-based perceptron hereafter, that can pave the way for constructing multilayer neural networks for generating nonlinear outputs.

### 2.2 Design and Analysis of a Sigma-Based Perceptron with ASReLU Activation Function and Sharing Limited Resources

**Figure 3:**
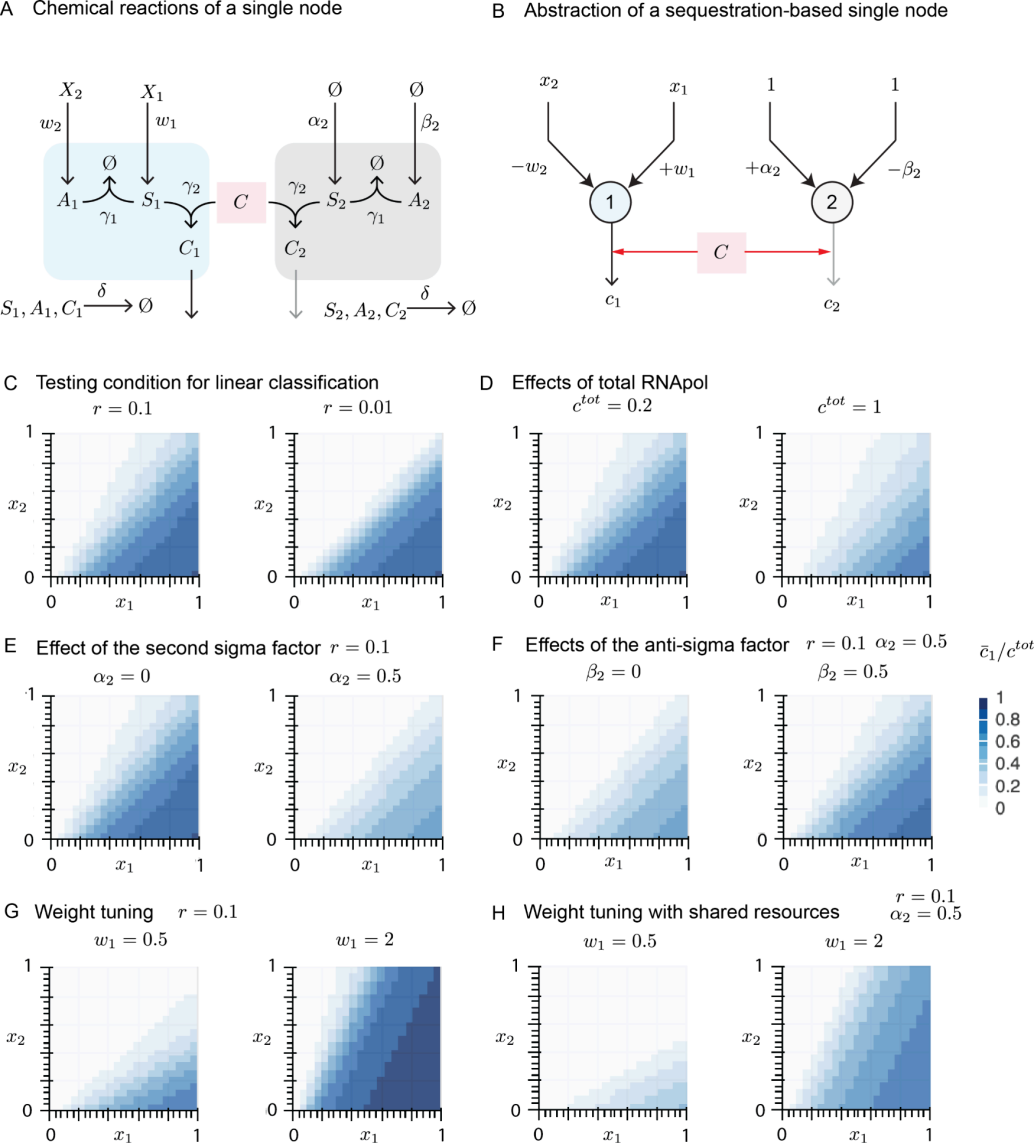
Sigma-based biomolecular network with thresholding response demonstrate characteristics of a perceptron. **A**: Chemical reactions represent a single perceptron in the presence of a competing node. See Table 1 for the value of input weights. **B**: The equivalent representation of the chemical reactions in the context of artificial neural networks. **C**: Linear decision boundary of a single node in the absence of competition. Lower *r* demonstrates linear decision boundary while higher *r* generates linear decision boundaries with weights that vary as a function of input. **D**: Linear decision boundary of a single node with different amounts of total *C* in the absence of competition is illustrated. A higher concentration of total *C* allows a higher range of responses and a lack of saturation observed with lower *c*^tot^. **E**: Linear decision boundary of a perceptron in the presence of a competing node. α_2_ represents the concentration of species that produces competing *S*_2_. A higher amount of *S*_2_ reduces the available concentration of total *C*, thus suppressing the response of the decision boundary while keeping its pattern intact. **F**: The effect of concentration of *A*_2_ (controlled by β_2_), which sequesters competing *S*_2_, on the linear decision boundary of a perceptron in the presence of limited resources and a competing node. Higher sequestration yields a higher available concentration of total *C*, thus increasing the amplitude of the perceptron response. **G**: Linear decision boundary of a single node in the absence of a competing factor can be tuned by adjusting the weight of the input even when *r* = 0.1. **H**: Tuning the linear decision boundary of the perceptron in the presence of a competing node can still be realized by changing the input weight. The amplitude of the response, however, is suppressed due to the lower availability of *C*.

After confirming the output of the sigma-based perceptron with an ASReLU activation function, we sought to determine whether this system demonstrates typical characteristics of a single node or perceptron in a neural network in the presence of a competing node. We test various input ranges of the perceptron and its competing counterpart to investigate the perceptron’s linear decision boundary as well as weight-tuning for manipulating its decision boundary. Thus, we model the binding of a sigma factor (*S*_1_) to RNApol in the presence of its antisigma (*A*_1_) as well as a competing sigma-antisigma pair (*S*_2_ and *A*_2_) and look at the steady-state total amount of RNApol bound to *S*1 (denoted as *c̅*_1_) as the output of the node in response to a wide range of inputs (Figures 3A and B).

First, we investigate the output of the single node in isolation (without competing factors) to understand the effect of binding kinetics between *S*_1_ and *A*_1_ (γ_1_) as well as *S*_1_ and *C* (γ_2_) on the perceptron’s decision boundary. Our findings show that the competitive binding ratio plays a critical role in determining the response pattern or the decision boundary of the perceptron. Equation 4 below provides insight into how *r* and total resources *c*^tot^ affect the decision boundary (see SI section 1.2 for derivation):
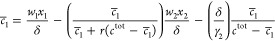
4

The last term in eq 4 simply introduces a bias to the decision boundary which only influences the response amplitude while leaving the response pattern, dictated by weights *w*_1_ and *w*_2_, intact. Additionally, the coefficient of *x*_2_ depends on both variables *w*_2_ and . Therefore, the competitive binding ratio, depending on its magnitude, can change the slope of the decision boundary. When *r* → 0 (Figure 3C, right), the coefficient of  in eq 4 converges to 1. Hence, in this regime, the pattern of decision boundary remains linear across the input ranges, although its bias changes depending on the inputs. However, as *r* becomes greater, the effect of limited resources on the decision boundary strengthens (Figure 3C, left). In such a condition, the decision boundary still remains linear, but since *c̅*_1_ is a function of *x*_1_ and *x*_2_, the slope of the decision boundary varies across the range of the inputs as an inverse function of *c*^tot^ – *c̅*_1_. Intuitively, when *r* → 0, the sequestration rate γ_1_ is much higher than that γ_2_. Thus, the available amount of *S*_1_ becomes equal to *x*_1_*w*_1_ – *x*_2_*w*_2_ which corresponds to an excess amount of *S*_1_ after binding to *A*_1_. In this case, regardless of the binding rate between *S*_1_ and RNApol *C*, the output will be a simple linear function of excess *S*_1_. On the other hand, when the sequestration rate γ_1_ is slow and comparable to the binding rate between *S*_1_ and *C* (γ_2_), the dynamics of binding between *S*_1_ and *A*_1_ in conjunction with binding of *S*_1_ and *C* dictates nonlinearity on the output of the sequestration and complex formation reactions (seen in Figure 2F). The decision boundary of the perceptron consequently consists of linear decision boundaries with slopes that change as the inputs vary. Nevertheless, as long as *r* ≪ 1, the perceptron still functions similarly to the ideal condition (when *r* → 0) and generates a decision boundary which resembles the ideal linear condition and can be used for the construction of more complicated architectures.

Importantly, in the construction of our model, we assumed that the sequestration species *S*_1_ and *A*_1_ are generated from an orthogonal RNA polymerase. i.e., production of *S*_1_ and *A*_1_ does not consume shared resources *C*. Such a scenario corresponds to systems in which input species promote the production of *S*_1_ and *A*_1_ through polymerases such as T7 or SP6 RNA polymerase (we call this scenario an uncoupled input–output relationship) instead of a housekeeping sigma factor (e.g., sigma 70. We call this scenario coupled input–output relationship). To investigate the effect of resource consumption by input species (i.e., if *S*_1_ and *A*_1_ are generated by complexes of housekeeping sigma factors with core RNAPol), we modified our model to include coupling between the resource consumption of *S*_1_ and input species *X*_1_ and *X*_2_ (Figure S2A). Assuming that housekeeping sigma factor *S*_*C*_ is responsible for the production of *S*_1_ and *A*_1_ via *S*_*C*_–*C* complexes denoted as *C*_*S*_ and *C*_*A*_, respectively, our model shows the effect of resource consumption by *S*_*C*_ in different input regimes (Figures S2B and C). Specifically, when [*x*_1_ → 1, *x*_2_ → 1], both *C*_*S*_ and *C*_*A*_ use the limited resource *C*, thus causing a reduction in the production amount of sigma factor and antisigma protein compared to the uncoupled case. Consequently, this effect leaves less resources for *S*_1_. Therefore, the response magnitude of the perceptron with a coupled input–output relationship becomes lower than the uncoupled case while the decision boundary remains similar (Figures S2D and E). Therefore, while the perceptron still demonstrates the linear decision boundary when input species consume limited resources, sharing limited resources between the perceptron sigma factor and the perceptron inputs suppresses the output magnitude. Therefore, in practice, production from input species through orthogonal RNA polymerases like T7 and SP6 would be preferred, as it circumvents the limitations of the input–output coupling.

Next, we investigate how the total amount of RNApol, *C*, affects the perceptron function. Since *C* does not play a role in the dynamics of the ASReLU function, its variation reveals itself as a simple increase or decrease in the perceptron response amplitude (Figure 3D). This effect occurs because the normalized output of the ASReLU, *c̅*_1_^*n*^, is completely independent of *C* and varies only by *x*_1_ and *x*_2_ (see eq (23) in the SI). Therefore, a single perceptron node in the absence of any competing sigma factors and in the presence of limited resources still acts as a linear classifier. However, the dynamic effects of antisigma–sigma binding on the system effectively change the weights of the perceptron decision boundary. Such changes, however, are minimal when *r* ≪ 1.

So far, we have focused on the decision boundary of the biological perceptron made by a sigma factor and its corresponding antisigma protein. Next, we consider whether the presence of another sigma factor (*S*_2_) and its antisigma protein (*A*_2_) changes the pattern of the perceptron decision boundary. Equation 5 provides an expression for the output of the perceptron as a function of inputs, *c̅*_1_, and *c̅*_2_. (See SI section 1.3 for derivation.)
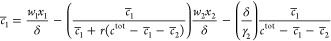
5

Equation 5 can be interpreted as an alternative form of eq 4 if *c*^tot^ in eq 4 is replaced with *c*^tot^ – *c̅*_2_. In other words, since the presence of a competing perceptron shrinks the amount of available resources, it amplifies the bias introduced to the perceptron output (by lowering the magnitude of the denominator in the last term in eq 5) and strengthens the input-dependent slope variation in decision boundary of the main perceptron (by increasing the coefficient of  in eq 5). Note that *c̅*_1_ in eq 5 is a function of inputs *x*_1_ and *x*_2_. Therefore, the magnitudes of introduced bias and change in the slope of the decision boundary will be varied in different input regimes. However, if *r* ≪ 1, the effect of competition and resource sharing on tuning decision boundary becomes negligible as the coefficient of  in eq 5 converges to 1.

Our simulations for a perceptron in a fast competitive binding regime over a range of different input concentrations verify our mathematical analysis by demonstrating that in the presence of another sigma factor (produced by input α_2_) competing to bind to the RNApol, the perceptron response is suppressed due to the bias introduced by the competing perceptron which lowers the amount of available RNApol (Figure 3E). The response pattern, on the other hand, remains mainly intact due to the low competitive binding ratio.

We also investigate how antisigma *A*_2_ (produced by input β_2_) influences the response of the perceptron in a resource-sharing system. Binding of *A*_2_ to *S*_2_ disables *S*_2_ from binding to RNApol, thereby increasing the total amount of RNApol available for *S*_1_ to bind. Therefore, we expect that the introduction of *A*_2_ to the system suppresses the bias effect of *S*_2_ on the perceptron response pattern (last term in eq 5). Indeed, our simulations show that an increase in the production of *A*_2_ increases the perceptron output (Figure 3F), thus confirming our hypothesis.

Our mathematical analysis of the perceptron output in the presence of a competing perceptron leads to an expression for *c̅*_1_ that is dependent on inputs and *c̅*_2_ (eq (48) in the SI), indicating that kinetics of binding between *S*_2_ and *A*_2_ would reveal its effect on perceptron output by simply varying its response amplitude while leaving its response pattern intact as long as the perceptron functions in the fast competitive binding regime. (See SI section 1.3 for mathematical derivation. See Figures S3 and S4 for simulations over a wide range of different *S*_2_ and *A*_2_ concentrations and different kinetics of *S*_2_–*A*_2_ binding, respectively.)

Lastly, as weight tuning is a fundamental characteristic of nodes in neural networks that defines their individual decision boundaries, we determine the feasibility of tuning the weights applied to our biological perceptron in the presence or absence of resource sharing. We first analyze an individual node with a limited amount of *C* and observe the change in the output pattern as *w*_1_ increased (Figure 3G). Similar to perceptrons used in ANNs, adjusting the weight applied to the input results in a discernible change in the slope of the response pattern, which is in agreement with our mathematical analysis (see 1.3 in SI). Aligned with our expectation, introducing a second node *S*_2_ that imposes resources sharing on the system preserves the weight-tuning characteristic of the perceptron and only affects its response amplitude by lowering the ASReLU saturation limit (Figure 3H).

In addition to steady-state analysis, we investigated the transient response of the sigma-based perceptron to study its function before it reaches its stable decision boundary. We observed that the perceptron output reaches the steady state shortly after the beginning of the simulation regardless of the parameters defining the concentration of species or the kinetics of the sequestration reaction (Figure S5A). Additionally, the perceptron decision boundary forms even in the transient state (Figure S5B). While in all scenarios with different values of α and β in either slow or fast sequestration regimes, the decision boundary is similar to the steady-state response pattern (i.e., maximum response generation at [*x*_1_ → 1, *x*_2_ → 0]), the response magnitude is smaller than the steady-state response.

Taken together, our simulations elucidate the effect of a competing sigma factor on the perceptron output magnitude in the fast competitive binding regime and also demonstrate the weight-tuning ability of perceptron with or without resource sharing. Therefore, we concluded that by operating in a fast competitive binding regime, sequestration-based perceptrons can demonstrate linear decision boundaries despite sharing limited resources which in turn allows the construction of multilayer perceptron networks.

### 2.3 Biomolecular Neural Networks Generate Nonlinear Classifiers in the Presence of Shared Resources

While a single sigma-based perceptron generates a tunable decision boundary even with sharing limited resources (Figure 3), most biologically relevant biocomputation processes such as competitive ligand binding and protein dimerization generate sophisticated nonlinear responses that rely on the protein–protein interactions and the concentration of competing dimerizing proteins or ligands.^23,24^ Therefore, we aim to utilize the sigma-based perceptron as a basic building block of more intricate networks made of multiple perceptrons that are capable of generating nonlinear outputs.

**Figure 4:**
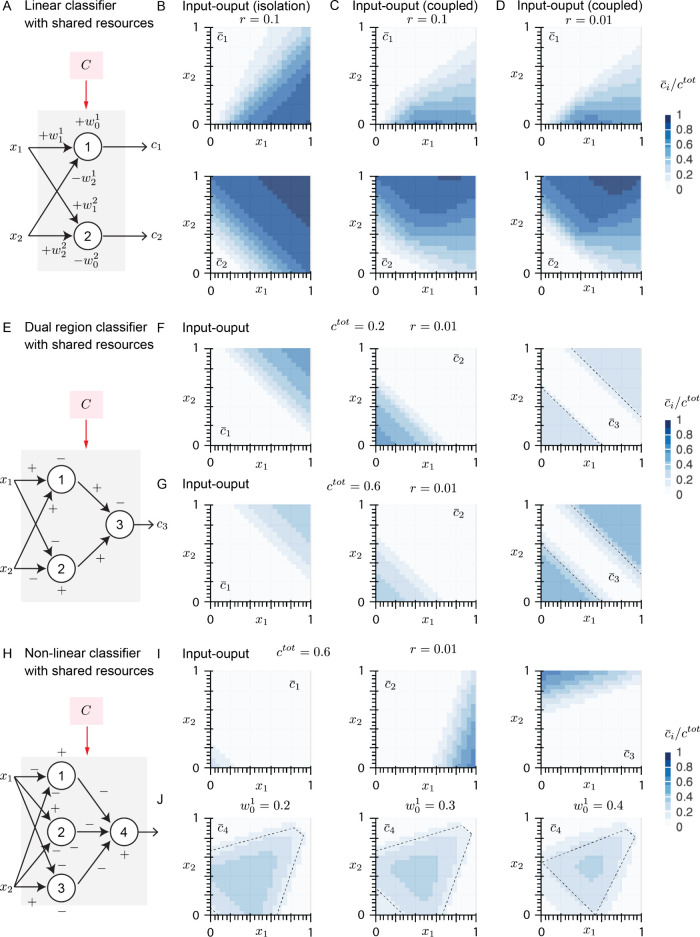
Biomolecular neural networks enable implementation of linear and nonlinear classifiers: **A**: Schematic representation of a single-layer neural network that generates a linear classifier. Both perceptrons share limited resources *C*. See Table 1 for the value of input weights. **B**: Linear decision boundary of perceptron 1 (top) and perceptron 2 (bottom) in the absence of competitive binding. **C**: Decision boundaries of perceptrons 1 (top) and 2 (bottom) when perceptrons compete to bind to the limited resources *C*. The competitive binding ratio *r* is 0.1. **D**: Decision boundary of perceptrons 1 (top) and 2 (bottom) when perceptrons compete to bind to the limited resources *C*. The competitive binding ratio *r* is 0.01. Competitive binding introduces nonlinearity to the classifier by reducing the output amplitude in input regimes in which both perceptrons require resources. **E**: Schematic representation of the architecture of the dual region classifier multilayer neural network with shared resources. + and – signs indicate the weights of inputs and whether they promote the expression of sigma or antisigma proteins. See Table 1 for the value of input weights. **F**: Output maps of the perceptrons in the first layer (left and middle for *C*_1_ and *C*_2_, respectively) each represent a linear classifier with minimal interference. Right panel depicts network output (*c*_3_) which comprises the dual region classifier with *c*^tot^ = 0.2. The dashed lines represent the decision boundary of an ideal perceptron with the same weights which function in the absence of resource constraints. **G**: Output maps of the perceptrons in the first layer (left and middle) and network output (right) comprising the dual region classifier with *c*^tot^ = 0.6. The pattern of the classifier is invariant to *c*^tot^ while its amplitude varies with *c*^tot^. The dashed lines represent the decision boundary of an ideal perceptron with the same weights which function in the absence of resource constraints. **H**: Schematic representation of the multilayer neural network with a nonlinear classifier output. + and – signs indicate the weights of inputs and whether they promote the expression of sigma or antisigma proteins. See Table 1 for the value of input weights. **I**: The output of perceptrons in the first layer of the network are depicted. Perceptrons 1 (left), 2 (middle), and 3 (right) each recapitulate a linear classifier with zero to little interference in their linear decision boundaries. **J**: The output of the network depicted in **H** (*c*_4_) is a nonlinear classifier. The classifier boundary can be tuned by adjusting the biases of the nodes in the first layer. Shown are examples of different decision boundaries by changing the bias of perceptron 1 to 0.2 (left), 0.3 (middle), and 0.4 (right). For each case, the dashed lines represent the decision boundary of an ideal perceptron with the same weights which functions in the absence of resource constraints.

First, we investigate a two-node network where each node representing a sigma-based perceptron receives the two inputs with unique weights (Figure 4A). This simple network allows us to study the effect of sharing limited resources on the output of nodes in the same layer (see SI section 2.1 for mathematical representation of the network). We note that the output of each node or perceptron represents the molecular complex made by binding of the perceptron sigma factor to the shared resources RNApol (as depicted in Figure 2D) which in turn can promote expression of another sigma factor (corresponding to positive weight) or another antisigma molecule (corresponding to negative weight). Therefore, *c*_*i*_ stands for the complex between *S*_*i*_ and *C* as the output of each node in the networks illustrated in Figure 4.

In the absence of resource sharing, each node’s output is a linear combination of its inputs (Figure 3C). Therefore, with the given weights denoted in Figure 4A, linear patterns for outputs of nodes 1 and 2 are observed (Figure 4B). However, when the binding competition between sigma factors is taken into account, the effect of limited resources and competition on the network output is elucidated as the attenuation of outputs for each node in certain input regimes (Figure 4C) resulting in a nonlinear response pattern. Notably, the response of each node is maximally affected, where the competing node is consuming most of the resources. For instance, in isolation, the output of node 2 (*C*_2_) has its highest expression level when [*x*_1_, *x*_2_] → 1 (Figure 4B, bottom). Consequently, the response pattern of *C*_1_ is highly diminished in that region (Figure 4C) due to the effect of resource sharing with node 2. In a similar fashion, the output pattern of *C*_2_ demonstrates its highest attenuation where *C*_1_ is expressed the most in isolation (*x*_1_ → 1, *x*_2_ → 0), thus leaving a smaller amount of available RNApol (*C*) for the production of *C*_2_. Hence, the concerted effect of resource sharing in a wide range of inputs imposes nonlinearity on the output of nodes in the same layer due to the emergence of a dominant perceptron that consumes most of the resources. This effect can also be deduced from eq 5 with the knowledge that both *c̅*_1_ and *c̅*_2_ are functions of *x*_1_ and *x*_2_. Therefore, in certain input regimes where either *C*_1_ or *C*_2_ is strongly expressed, the effects of competitive resource sharing detailed in the previous section become strengthened.

Interestingly, the network output is influenced differently in the fast and slow competitive binding regimes. In a fast competitive binding regime, *c̅*_1_ and *c̅*_2_ are linear functions of *x*_1_ and *x*_2_. Therefore, the outputs *C*_1_ and *C*_2_ display sharper deviations when both are dependent on inputs (Figure 4D) due to the stronger effect of the bias term in eq 5 imposed by sharp reduction of resources (see Figure S6 for isolated perceptron responses in fast competitive binding regime). Overall, we conclude that resource sharing can significantly induce nonlinearity to the network output pattern and the highest impact of competitive resource sharing reveals itself where the response of isolated nodes overlap with each other, corresponding to input regimes with maximum resource sharing.

Knowing that overlapping responses can cause nonlinearity in the network outputs, we next consider whether we can still implement nonlinear networks with decision boundaries that are distorted minimally due to resource sharing. We reason that if the outputs of the same layer do not overlap with each other, the overall output of the network will follow the design principles dictated by the input weights and will not have undesired nonlinearity. To test this hypothesis, we look at a simple network made of three nodes that reconstitutes a dual region classifier (band-stop filter) represented in Figure 4E (see SI section 2.2 for mathematical representation of the network and Figure S7 for the ideal response of this network in the absence of sharing limited resources). In order to avoid nonlinearity induced by resource sharing, we design the network such that the node outputs have minimal overlap with each other (Figure 4F, left and middle panels). We expect that the lack of overlap between node outputs would prevent unwanted nonlinearity in the network output (*C*_3_). Aligned with our expectation, our simulations demonstrate the network response consisting of two separate linear regions despite resource sharing (Figure 4F, right). We also confirmed that an increase in the amount of available resources (*C*) decreases the effect of competition for resources and does not alter the decision boundary of first layer nodes (Figure 4G, left and middle) as well as the overall network (Figure 4G, right). Therefore, we conclude that linear responses can be engineered in a network, despite the presence of resource sharing, by tuning input weights such that the outputs of the nodes in the same layer do not overlap.

Finally, we seek to create a biomolecular neural network that generates a nonlinear classifier. Such nonlinear outputs are key features of many biological processes and drivers of cell decision-making.^56−58^ We design a simple network with specific input weights based on the interaction of multiple sigma factors and their corresponding antisigma molecules that generates a nonlinear classifier resembling a band-pass filter (Figure 4H, see SI section 2.3 for ODEs describing the network). As in the previous design, we want the outputs of the first layer to have minimal overlap with each other to avoid nonlinearity induced by resource sharing. Our simulations depict that the first layer outputs *C*_1_, *C*_2_, and *C*_3_ have minimal overlap with each other given the particular weights and biases (Figure 4I). Consequently, our simulations demonstrate that network output (*C*_4_) creates the nonlinear classifier (Figure 4J) that is aligned with our ideal design expectations (Figure S8). Consistent with our findings in the previous section, changing the *c*^tot^ does not change the nonlinear pattern of the decision boundary but tunes its amplitude (Figure S9).

We also look at the effect of input bias on the first layer of output (Figure 4J). Indeed, in accordance with the ideal design (Figure S8), increasing the bias of node 1 amplifies its steady-state output (*c̅*_1_) which subsequently further sequesters the amount of available *S*_4_, thus suppressing the network response in its corresponding input regime ([*x*_1_, *x*_2_] → 0) without significant deviation from design expectations (Figure S8). Therefore, we showed that even in the presence of resource sharing, we can develop nonlinear biological neural networks to realize dual region and nonlinear classifiers. We also demonstrated that we can modulate the network response by tuning the model parameters, which are independent of limited resources.

## 3 Discussion

Biological signal processing units that reconstitute molecular linear and nonlinear classifiers are powerful tools that enable cellular decision-making, precise cell programming, highly discriminatory input processing, and ultrasensitive molecular biosensors for applications such as CAR T-cell engineering or *in vitro* diagnostics. A biological perceptron ideally allows linear classification while a combination of biological perceptrons can create biomolecular neural networks that compute nonlinear classification.

Among different approaches that are used to construct biochemical neural networks and classifiers, sequestration-based networks are of significant interest because of their simplicity and compatibility with *in vivo* systems.^29,36^ However, the effect of physiological constraints such as a limitation on resources, as well as competitive binding between elements of molecular classifier networks, specifically sequestration-based networks, on the classification function, has remained unexplored.

We mathematically modeled sequestration-based biochemical neural networks and investigated how sharing limited resources, a ubiquitous feature of physiological systems, influences the function of the neural network decision boundary. We chose a network of sigma factors, their corresponding antisigma proteins, and core RNA polymerase as our model system. Our analyses demonstrated that a single perceptron, the basic building block of neural networks, with a ReLU-like activation function is recapitulated by modeling the interaction of one sigma factor with its antisigma molecule. We further showed that modifying the model to include a limited amount of core RNApol in the system changes the activation function of the sigma-based perceptron to an asymptotic saturated ReLU. Drawing inspiration from natural systems where multiple sigma factors compete to bind to a limited pool of RNApol, we altered the model to include another sigma factor and found that the decision boundary of the sigma-based perceptron remains the same, although its output is suppressed.

While conditions of biochemical *in vitro* reactions are primarily controlled, in living organisms, endogenous factors can cause perturbations and deviations from ideal design. For instance, in bacteria, although a certain sigma factor might be designed to control gene expression, multiple other sigma factors compete with the engineered sigma factor to bind to a limited amount of RNApol. To include competitive binding to shared resources, we increased the number of perceptrons, controlled by the same inputs, to two. We found that in specific input regimes where the outputs of the perceptrons interfere with each other, one dominant perceptron emerges and consumes most of the resources whereas the decision boundary of the nondominant perceptron significantly deviates from its ideal design.

Given that engineering any kind of nonlinear response by neural networks requires multilayer perceptrons, we investigated conditions where despite resource sharing, nonlinear decision boundaries could be designed. Knowing that interference of outputs of perceptrons in the same layer raises deviations of the linear decision boundary, we engineered particular multilayer perceptrons in which nonlinear decision boundaries were successfully demonstrated. We showed that despite sharing limited resources, dual region and nonlinear classifiers resembling band-stop and band-pass filters can be implemented in sigma-based neural networks using different architectures of 2-layer neural networks with minimal deviation from ideal design.

Since sharing limited resources is not exclusive to the sigma-based neural networks, our findings can be extended to other biocomputational input-processing systems used in living cells. Taking Clustered Regularly Interspaced Short Palindromic Repeats (CRISPR) gene editing as an example, the sequestration of a single guide RNA (sgRNA) strand by its complementary RNA, or antiguide RNA, is analogous to the interaction of a sigma factor with its antisigma protein. If the sgRNA is engineered to drive a CRISPR reaction, then the Cas protein will consequently be the limited resource to which all sgRNAs will compete to bind to. Similarly, a limited pool of proteolytic substrates that are catalyzed by a protease-based neural network^38,62^ will follow the functional principles outlined in this work. Therefore, the principles of input classification under the effects of sharing limited resources outlined in this paper can be extended and used for the design of other molecular classifiers.

An advantage of sigma-based sequestration-based neural networks over DNA-based neural networks is that they originate from endogenous proteins in bacteria. Therefore, sigma-based neural networks can be implemented both in bacterial systems as well as bacterial lysate-based cell-free expression systems, thus expanding their applications as living computers as well as *in vitro* biosensors and synthetic cell-based biocomputers.^63,64^ To indicate the possible implementation of neural networks demonstrated here (Figure 4), we present schematics of required DNA sequences for each network in Figures S10 and S11. Given that construction and function of sigma-based perceptron relies on transcription-translation as well as protein degradation, the perceptron parameters (i.e., input weights), which collectively control the whole network behavior, can be determined with common tools of tuning genetic circuits.^4,65,66^ For example, changing the strength of ribosome-binding site or promoters that control expression of sigma and antisigma proteins can tune the slope of the perceptron decision boundary. Other controllable elements such as plasmid copy number (changed by switching origin of replication), degradation rate (changed by addition of degradation tags to the perceptron proteins), or sequestration rate (tuned via mutagenesis in sigma and antisigma proteins) may regulate the function of perceptron by effectively changing its weights as well, the practical demonstration of which awaits future studies.

Additionally, unlike neuromorphic systems made of inducible genetic circuits, in sequestration-based networks, both positive and negative weights can be engineered, thereby making them more flexible and applicable for generating nonlinear response patterns. Since outputs of sigma-based perceptrons are transcriptionally active sigma-RNApol complexes, they can be programmed to drive the expression of other sigma factors. Relying on this characteristic of sigma-based perceptrons, multilayered perceptrons that are capable of recapitulating sophisticated nonlinear response patterns can be designed readily. However, as shown in this work, for the neural network to function as designed and avoid perturbations caused by sharing limited resources among perceptrons, the outputs of perceptrons in the same layer must have minimal interference.

Although using sigma factors, which are endogenous molecules in bacterial systems, as components of a biocomputational module offers flexibility and simple implementation, we note that utilizing sigma factors as building blocks of BNNs could face challenges originating from their native roles in physiology of bacteria. Overexpression of endogenous sigma factors may affect native biological processes in bacteria, cause sigma factor crosstalk with other proteins, or introduce growth issues. Of these, unintentional binding of sigma factors to other proteins could specifically change the behavior of BNN due to unwanted perturbations that effectively change the weights of the network. An avenue to explore in order to circumvent this challenge is to use orthogonal exogenous sigma factors to construct the BNN. Exploiting exogenous sigma factors would prevent unwanted binding of perceptron sigma factors to native molecules as well as interference with biological events in bacteria. In fact, sequestration of *Bacillus subtilis* sigma and antisigma proteins sigW and rsiW, respectively, was previously used in *E. coli* for construction of a closed loop biomolecular integral feedback control circuit.^51^ Interestingly, the output of this circuit resembles the behavior of a sigma-based perceptron presented in this study, thus promising the potential of exogenous sigma factors as building blocks of BNNs. Further, the challenges of using endogenous sigma factors may be less problematic when BNNs are implemented using *in vitro* bacterial-cell-free expression systems due to the absence of endogenous biological processes.

While we demonstrated designs of linear and nonlinear classifiers in this work with predetermined weights that generate desirable decision boundaries, we note that sigma-based neural networks are not capable of learning through common algorithms like backpropagation. i.e., the input weights that directly determine the decision boundary are chosen by the designer. However, by utilizing the mathematical analysis presented here, one can implement optimization approaches such as particle swarm optimization or other heuristic algorithms to find the appropriate weights for the generation of the desired decision boundary *in silico* prior to testing them *in vivo* or *in vitro*. Such optimization algorithms can also be implemented to expand the function of BNNs to regimes where nonlinear effects of resource constraints or perturbations due to nonspecific or unwanted bindings described above appear. Therefore, developing BNNs with biological resource constraints capable of recognizing any arbitrary input pattern using optimization methods is an avenue worth exploring in the future.

Recently, it was shown that many cancer cell types can be recognized with higher precision if a combination of two antigens is used to identify them instead of traditionally using one biomarker.^67^ Implementing nonlinear output patterns with sequestration-based neural networks could increase the recognition ability of engineered cellular systems like CAR T-cells by equipping them with information-processing neural networks that generate desired outputs only in designed antigen concentration regimes. Similarly, by coupling inputs to the expression of sigma factors and their antisigma molecules, more sensitive, precise, and versatile *in vitro* biosensors for the detection of pathogens, substances, and biomarkers can be constructed.

With the nanofabrication technology in the semiconductor industry approaching its physical limits of manufacturing smaller and smaller elements,^68^ alternative computational devices with biological components are gaining increasing interest. However, current biocomputational systems are only in their infancy. Although biocomputation in living systems was initially shown more than two decades ago using genetic circuits, the limited range of computational tasks that genetic circuits can perform as well as the digital nature of their input-outputs make their application limited. With the recent booming advance of AI, biochemical approaches that recapitulate biological neural networks holding the potential to perform intricate computational tasks have gained attention.^3,69^ Our study provides a general framework for designing biological perceptron or linear classifiers using existing biomolecular tools in the presence of resource constraints that are ubiquitous in physiological conditions. This framework, thus, is the first step toward designing sophisticated biomolecular neural networks that equip engineered cells with high-level computational and decision-making abilities.

In addition to transformative applications of sigma-based neural networks used in a forward-engineering manner in both cellular and cell-free systems, the fact that sigma and antisigma molecules can construct complicated computational modules elucidates the hidden capabilities of these rather simple transcriptional regulation molecules in cells. It was revealed by Park et al. that sigma factors share the resources in a pulsatile manner.^18^ However, here we demonstrated that sharing limited resources influences sigma-based processes beyond time sharing. Bacterial cells have many sigma factors, some of which are activated only when their inputs meet certain conditions. However, how bacteria utilize the principles of molecular sequestration, as well as sharing limited resources to respond differently to input combinations, awaits future studies. Additionally, if bacteria are able to process certain input patterns using their endogenous sigma-based neural networks, the nature of these patterns and their role in guiding bacteria to make particular decisions remain unclear. In conclusion, our investigation demonstrates the effects of resource-sharing on sigma-based sequestration-based neural networks with up to four sigma factors and provides an outline for designing sigma-based nonlinear neural networks in bacterial systems.

## 4 Methods

Sequestration and binding reactions were modeled into ODEs using the law of mass action. See SI for the derivation of ODEs describing sigma-antisigma molecular sequestration and the construction of a perceptron based on their behavior. Where possible, analytical expressions were derived for steady-state outputs of the reactions. All ODEs were modeled, and the steady-state solutions were solved by a custom Python code using the numpy and scipy packages. ODEs were integrated using odeint function from the scipy package to obtain transient and steady-state response of reactions. The figures were plotted using the Bokeh and Matplotlib packages. The codes used for solving the ODEs and generating figures can be found here: GitHub - mhossein7/BNN_shared_resources

## Supporting Information Available

Derivation of ODEs describing sigma-antisigma molecular sequestration at the steady state and the construction of a perceptron based on their behavior (PDF)
